# Recent Incidence of Human Malaria Caused by *Plasmodium knowlesi* in the Villages in Kudat Peninsula, Sabah, Malaysia: Mapping of The Infection Risk Using Remote Sensing Data

**DOI:** 10.3390/ijerph16162954

**Published:** 2019-08-16

**Authors:** Shigeharu Sato, Bumpei Tojo, Tomonori Hoshi, Lis Izni Fanirah Minsong, Omar Kwang Kugan, Nelbon Giloi, Kamruddin Ahmed, Saffree Mohammad Jeffree, Kazuhiko Moji, Kiyoshi Kita

**Affiliations:** 1 Faculty of Medicine and Health Sciences, Universiti Malaysia Sabah, Kota Kinabalu 88400, Sabah, Malaysia; 2 Nagasaki University School of Tropical Medicine and Global Health, Nagasaki 852-8523, Japan; 3 Institute of Tropical Medicine, Nagasaki University, Nagasaki 852-8523, Japan; 4 Kudat Health Office, Ministry of Health Malaysia, Beg Berkunci No. 6, Kudat 89059, Sabah, Malaysia

**Keywords:** *Plasmodium knowlesi*, infection risk map, geographical analysis, remote sensing, MODIS, EVI phenology, generalised linear mixture model, Bayesian inference

## Abstract

*Plasmodium knowlesi* (Pk) is a malaria parasite that naturally infects macaque monkeys in Southeast Asia. Pk malaria, the zoonosis transmitted from the infected monkeys to the humans by *Anopheles* mosquito vectors, is now a serious health problem in Malaysian Borneo. To create a strategic plan to control Pk malaria, it is important to estimate the occurrence of the disease correctly. The rise of Pk malaria has been explained as being due to ecological changes, especially deforestation. In this research, we analysed the time-series satellite images of MODIS (MODerate-resolution Imaging Spectroradiometer) of the Kudat Peninsula in Sabah and created the “Pk risk map” on which the Land-Use and Land-Cover (LULC) information was visualised. The case number of Pk malaria of a village appeared to have a correlation with the quantity of two specific LULC classes, the mosaic landscape of oil palm groves and the nearby land-use patches of dense forest, surrounding the village. Applying a Poisson multivariate regression with a generalised linear mixture model (GLMM), the occurrence of Pk malaria cases was estimated from the population and the quantified LULC distribution on the map. The obtained estimations explained the real case numbers well, when the contribution of another risk factor, possibly the occupation of the villagers, is considered. This implies that the occurrence of the Pk malaria cases of a village can be predictable from the population of the village and the LULC distribution shown around it on the map. The Pk risk map will help to assess the Pk malaria risk distributions quantitatively and to discover the hidden key factors behind the spread of this zoonosis.

## 1. Introduction

*Plasmodium knowlesi* (Pk) is a malaria parasite that naturally infects macaque monkeys in southeast Asia [1]. This parasite, which is transmitted by *Anopheles* mosquitoes, can infect humans and causes a zoonotic malaria in them. Although a naturally acquired human case of Pk was reported as early as in 1965 [2], Pk malaria has not been paid much attention to until recently. This was because Pk malaria has long been misdiagnosed as another human malaria caused by *P. malariae* (Pm). The appearance of Pk and Pm infecting the human erythrocyte are too similar to be distinguished if one only relies on microscopic observation [3,4]. When polymerase chain reaction (PCR) was introduced in diagnosis in the early 2000s, it was revealed that Pk malaria was significantly prevalent in the Malaysian states of Sabah and Sarawak in the northern Borneo Island [5,6]. Today, most of the malaria cases identified in Malaysian Borneo are of Pk malaria.

The pioneering works carried out in Sarawak have suggested that the transmission of Pk to humans occurs very close to or within a deep forest [5]. In the early 2010s, a systematic investigation in Pk malaria also started in Sabah, and early epidemiological studies carried out in the Kudat district, northern Sabah, suggested that the transmission of this zoonotic malaria seems to occur close to or inside the houses of the patients [7]. This is unique because Pk transmission to humans takes place mainly in the forest, even in other parts of the world like in Sarawak [8]. To explain the cause of this uniqueness, which is only found in the Kudat district, we need to keep gathering more information on the diseases.

It has been hypothesised that the rise of Pk malaria cases in Malaysia was caused by ecological changes, particularly by deforestation [9]. Nearly 80% of the land surface of Sabah and Sarawak was affected by commercial logging or the expansion of oil palm plantations between 1990 and 2009 [10]. From these perspectives, we analysed the time-series satellite images of MODIS (MODerate-resolution Imaging Spectroradiometer), and quantified Land-Use and Land-Cover (LULC) distributions, especially forests, in the Kudat Peninsula.

Deforestation caused by an expansion of oil palm plantations spreads near the high malaria risk villages. Thus, we focused on quantifying oil palm plantations that are suspected to be the main ecological factors determining the spatial distribution of Pk malaria endemic villages. The clinical record of Pk malaria cases in 2016/17 in the study area was collected. Superimposing the geographical distribution of the Pk malaria cases on the map, we demonstrate how the LULC affects the Pk malaria risk in the study area.

## 2. Materials and Methods 

**Figure 1:**
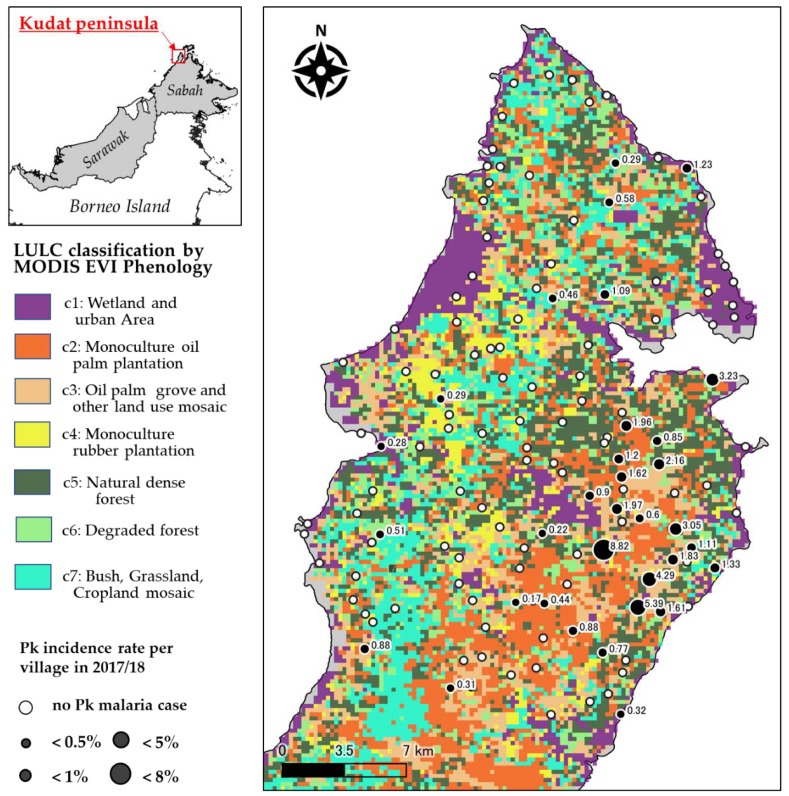
Maps of the study area. The left box shows the location of the Kudat peninsula in relation to Borneo Island. The right box displays the Land-Use and Land-Cover (LULC) classifications based on the MODerate-resolution Imaging Spectroradiometer Enhanced Vegetation Index (MODIS EVI) phenology. The locations and Pk malaria incidence rate (cases/village population) of the villages are also indicated. The black circles correspond to the villages with Pk malaria cases, and the size of each circle indicates the maximum incidence rate that was observed.

Ethical approval for this study was obtained from the Medical Research and Ethics Committee (MREC), Ministry of Health Malaysia (NMRR-18-3740-45271). The research area, the Kudat district, is a malaria endemic area. According to the census data, approximately 85,000 people lived there in 2010 [11]. In 2016/2017, a total of 107 Pk malaria cases amongst 54,759 people were reported from 133 villages within the Kudat Peninsula by the Kudat Health Office. These cases showed geographically accumulated patterns rather than scattered patterns (Figure 1). This implies that some spatial factors affect the distribution of Pk malaria cases in this area. Mosquitoes carrying the parasites are supposed to depend on the LULC surrounding them.

The LULC classification was classified based on the k-means method, using the pattern similarity of the time series of enhanced vegetation index (EVI) changes, phenology, of MODIS images. This phenology analysis allows us to distinguish the different types of vegetated land surfaces [12,13,14,15]. The MODIS has a relatively low spatial resolution (the pixel size is about 250 m) but a high observation frequency (image obtained every 1–2 days for everywhere in the world). Because of this short interval, the MODIS is suitable for the vegetation dynamics analysis. More specifically, high EVI values throughout the year can be observed in forest coverage, and EVI values in paddy fields show a unique variation according to agrarian cycles such as flooding, rice growing and water drainage.

The k-means algorithm is an unsupervised classification method in which the whole MODIS image pixels are statistically clustered into a given number of classes. For this reason, both the appropriate number of classes and the actual LULC of each generated class are unknown. Thus, we heuristically determined the number of classes by calculating the ratio of the within-cluster sum of squares for a clustering within the K cluster and the one within the K + 1 cluster, similar to applying Hartigan’s rule, which justifies the use of a K + 1 cluster when this ratio is greater than 10 [16]. The ratio was constantly reduced as the K number increased, until K = 33, after which the change in the ratio became small. Although the K at which the ratio first became less than 10 was 80, it is not realistic to use 80 image classes for the analysis. Therefore, the number of classes used for the LULC analysis in this study was determined to be 33.

When the phenology patterns (EVI waveform) of these 33 classes were compared, we found that the patterns of the EVI waveform of some classes were almost the same between them, though the level of the EVI waveform was different. Therefore, the 33 classes were integrated into 15 classes. Then, the integrated 15 classes were further simplified manually to 7 LULC classes, consulting a visual observation of Google Earth’s high-resolution satellite images. The 7 LULC classes finally obtained are: (c1) Wetland and urban area; (c2) Monoculture oil palm plantation; (c3) Oil palm grove and other land-use mosaic landscapes; (c4) Monoculture rubber plantation; (c5) Natural dense forest; (c6) Degraded forest; and (c7) Bush/Grassland/Cropland mosaic landscapes. Each LULC class was opted for via the visual observation of the Google Earth’s high-resolution satellite images. EVI 16-Day Global L3 Global 250 m data was used for analysis after a maximum-value two months composite (total 6 layers in 2017) was used to get rid of image noises resulting from cloud effects. 

In this study, the LULC within a 1.5 km radius from the centre of each village was used as the explanatory variable in a statistical modelling process. All of the classes included in the buffer were counted and converted (normalised) to 0–1 by a “pixel number/137 (maximum number)”, after which they were used for the model variables. This buffer radius was set in view of the flight range of several species of Anopheles mosquitoes, which varies between 1 and 3 km [17,18,19,20]. In this study, we presumed the area within the 500 m radius circle to be a residential area of villagers and the area between the 500 and 1500 m circles to be a surrounding environment that included the habitat of Anopheles mosquitoes. The population of each village was treated as an offset term. A Poisson multivariate regression was applied to estimate the Pk malaria cases (i.e., explained variable). First, a generalised linear model (GLM) was used for the estimation, after which a generalised linear mixture model (GLMM) was applied. In GLMM, the unknown group difference of each village was considered as a random factor. To estimate the parameters of GLMM, a Bayesian inference with Markov chain Monte Carlo method (MCMC) was adopted.

QGIS (version 3.2, 64 bit, QGIS Development Team, https://qgis.org) was used for the geographical analysis. A MODIS phenology classification and maximum likelihood estimations of GLM were performed in R (version 3.4.4, 64 bit, R Foundation for Statistical Computing, Vienna, Austria). The RStan (version 2.17.3, Stan Development Team, https://mc-stan.org) library for R was used for the MCMC sampling of GLMM, which requires a complex model fitting that takes the random effect into account.

## 3. Results

The results of the classification of the MODIS images revealed that the deterioration and disappearance of natural forests and the expansion of the oil palm plantations were progressing considerably in the Kudat Peninsula (Table 1). The natural dense forest was only about 1/5 of the area (19.4%). Meanwhile, a large-scale oil palm plantation, rubber forest, and degraded natural forest occupied 2/3 of the peninsula. The Kudat Health Office report confirmed that 107 Pk malaria cases were concentrated in the central eastern part of the peninsula, where large oil palm plantations spread. As the previous research suggested, the deforestation caused by the plantation expansion is likely to relate to the Pk malaria endemic.

**Figure 2:**
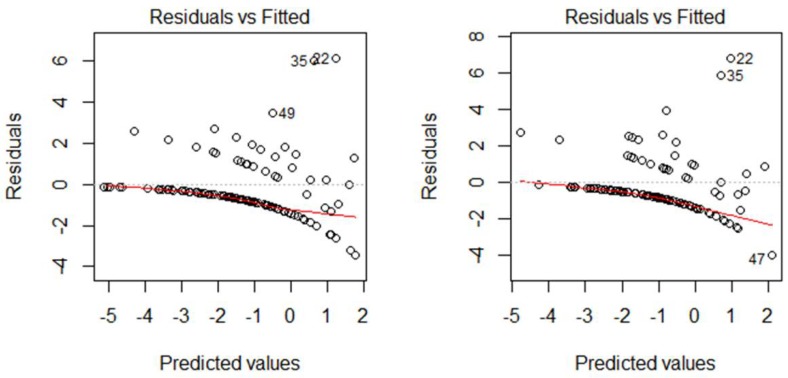
The Residuals vs. Fitted (Predicted) value plots of GLM Model 2 and Model 3.

The GLM examined the geographical relationship between the Pk malaria occurrence and the LULC distribution in a quantitative manner. All of the LULC classes of c1–c7 were included in GLM Model 1 (Table 2(1)). From GLM Model 1, three insignificant variables (c2; *p* = 0.25, c4; *p* = 0.06, c6; *p* = 0.55) were dropped to obtain a simplified GLM Model 2 (Table 2(2)). Furthermore, c1 and c7 were also removed from GLM Model 2 to obtain a further simplified GLM Model 3 (Table 2(3)). Although the Akaike information criterion (AIC) of GLM Model 2 was smaller than the one of GLM Models 3, Model 2 could not explain the higher Pk malaria cases in some villages well (Figure 2).

**Figure 3:**
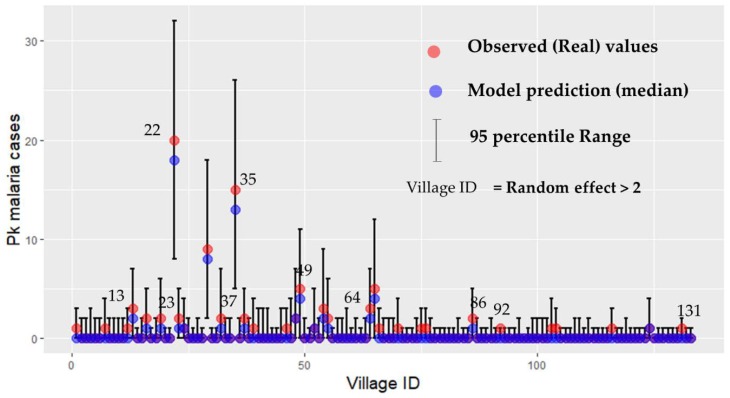
This figure compares the observed (real) cases of Pk malaria and the predicted (median), 95th percentile range of Pk malaria cases based on GLMM Model 3. The y axis is the Pk malaria cases and the x axis is each village ID from 1 to 133.

Following this, the explanatory variables of GLM Model 2 (c1 + c3 + c5 + c7) and GLM Model 3 (c3 + c5) were used in the GLMM. The product of twice the number of samples N and the widely applicable information criterion (WAIC) (2N × WAIC; equivalent to the AIC of a GLM model) in GLMM Model 2 and Model 3 showed a slight difference (model 2: 207.16; model 3: 209.37); however, both of them were significantly improved compared to the AIC of GLM models with the same parameters. Figure 3 shows the predicted Pk malaria cases of each village by GLMM Model 3 (c3 + c5) (95th percentile range), as well as the observed (real) values of Pk malaria cases. 

The GLMM Model 3 predicted that the Pk malaria risk is associated with the LULC classification of both (c3) the oil palm grove and other land-use mosaic landscapes and (c5) the natural dense forest near the village. Furthermore, the former factor of (c3) had nearly twice the number of Pk malaria cases (i.e., Pk malaria risk) compared to the later factor of (c5) (Table 3).

**Figure 4:**
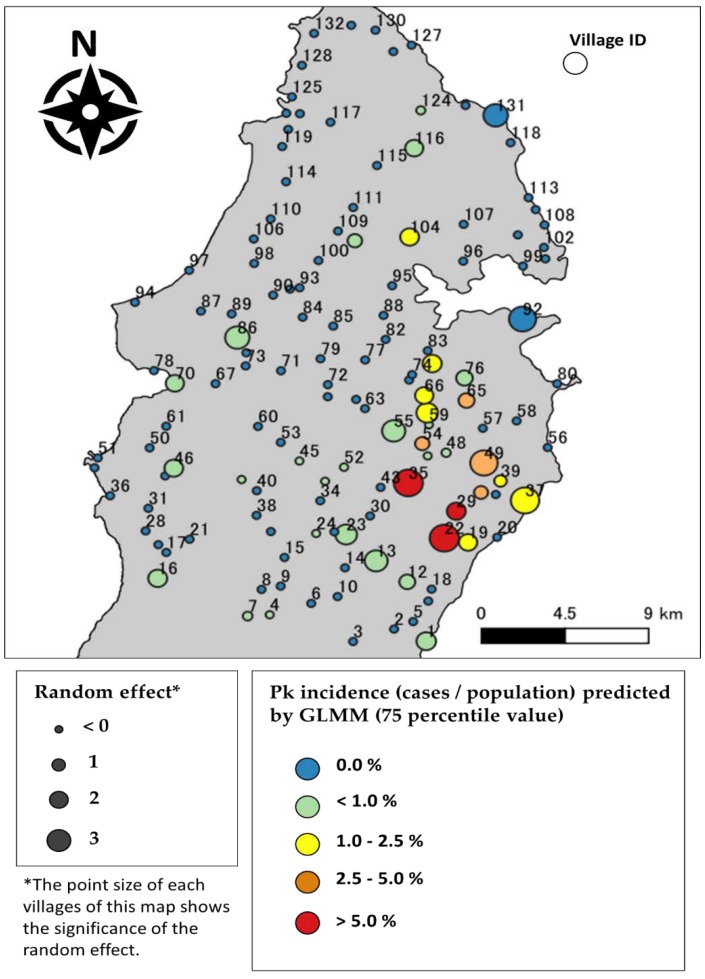
Pk malaria risk map of Kudat Peninsula. The map plots the generalised linear mixture model (GLMM) prediction value (75th percentile value of Markov chain Monte Carlo method (MCMC) sampling) of Pk malaria cases in each village. The Village IDs written on the map correspond to the Village IDs of Figure 3.

The results of GLMM Model 3 predicting Pk malaria cases were shown on the map (Figure 4). The map is almost consistent with the actual patient distribution shown in Figure 1. Moreover, the map suggested that the Pk malaria cases in each village were affected by the LULC surrounding the village, or by unknown group differences in each village. 

## 4. Discussion

In Malaysia, traditional human malaria caused by *P. falciparum* or *P. vivax* has been nearly eliminated because of an intensive control program rolled out throughout the country. However, Pk malaria remains difficult to control because the disease passes from the infected macaque monkeys living in the forest to the humans via mosquito vectors [6]. To design an effective strategy of Pk malaria control, it is important to reconsider the limitations of current malaria controls using long-lasting insecticidal nets (LLIN) and indoor residual spraying (IRS) [3]. Making disease risk maps is one of the effective uses of the growing Pk malaria reports. Such maps will be especially useful in rural areas where the available epidemiological information is limited. This approach of map creations in this study could be extended to villages for which only the coordinates are known. Therefore, no patient information is required, even though it is ideal to have such records. 

A recent published map of the estimated Pk malaria distribution is useful for planning optimised Pk malaria controls in southeast Asia [21]. However, satellite image-based approaches are prone to having a methodological challenge in classifying forest covers, particularly where natural forest and artificial plantations were jumbled together. In the case of Pk malaria, addressing this challenge was essential for proper disease risk estimations because the Pk malaria risk could be highly dependent on the LUCL. In this study, we succeeded in classifying seven LULC classes through the phenological analysis. This allowed us to consider the mosaic landscapes of oil palm groves, as well as other types of land-uses near patches of dense forest, where Pk malaria infections are more likely happen amongst macaques, malaria vector mosquitoes, and humans. 

Our final GLMM model (i.e., Model 3) estimated the relationship between Pk malaria and the LULC within a 1.5 km radius, and consequently allowed for the displaying of a Pk malaria risk map. The models followed the assumption that the Pk malaria vector density or Pk malaria infection risk changes based on the LULC. The model finesses of the GLM models were dramatically improved after considering the random effect of villages in the GLMM models. The role of the random effect was to control the unexpectedly higher numbers of Pk malaria cases in some villages. These unexpected or excessive Pk malaria cases might be associated with unknown factors, except for the LULC. One possible explanation for such factors could be occupations. 

We suspect that the random effect might be associated with the number of rubber tapping workers in each village. The rubber tapping workers are more likely to encounter mosquito bites than other villagers, since they tend to work outside from dawn to dusk, when *Anopheles* mosquitoes are most likely to bite humans. According to the clinical data in 2017 from the Kudat Health Office, 35% of patients (23/65) were involved in rubber tapping work. This detail was obtained in 25 villages from the highest malaria endemic area in the Kudat district. Other occupations were: student (10), farmer (9), no job (4), housewife (3), hunter (3), oil palm plantation worker (3), teacher (2), and others (8). Although oil plantation workers are considered to have a higher Pk malaria risk, rubber tapping workers may also have a higher risk.

## 5. Conclusions

The Pk malaria risk map shown here could be one of the useful guides for planning Pk malaria control. This map indicated a significantly high accuracy when comparing the estimated and actual reported cases. Despite the fact that the random effect included in the model remained uncertainty, this approach of creating maps could be extended to villages for which only the coordinates are known. Therefore, no patient information is required. We believe that our map could be of help for discovering the hidden key factors behind the spread of this zoonosis (e.g., it could be used to select study sites of the vector survey). 

## Figures and Tables

**Table 1:** Area coverage of each Land-Use and Land-Cover (LULC) in Kudat peninsula.

Class	LULC Classification	Area (km^2^)	%
c1	Wetland, Riverside Forest, Urban Area	82.7	11.5
c2	Monoculture Oil Palm Plantation	150.0	20.9
c3	Mosaic Oil Palm Planation	121.8	17.0
c4	Monoculture Rubber Plantation	46.3	6.5
c5	Dense Forest	140.2	19.6
c6	Degraded Forest	69.7	9.7
c7	Bush, Cropland mosaic	106.3	14.8
	Total	717.3	

**Table 2:** GLM Models.

**(1) GLM Model 1 (AIC: 350.5)**				
**Variables**	**Estimate**	**Std. Error**	**95% CI**	**Pr (>|z|)**
(Intercept)	−6.3777	1.1707	−8.67–−4.08	<0.001
c1	−8.2693	3.1856	−14.51–−2.03	0.0094
c2	−1.6270	1.4223	−4.41–−1.16	0.2527
c3	6.4465	1.2518	3.99–8.90	<0.001
c4	−4.0482	2.1634	−8.29–0.19	0.0613
c5	3.5766	1.4040	0.82–6.33	0.0109
c6	−1.6423	2.7802	−7.09–3.81	0.5547
c7	−4.4168	2.2582	−8.84–0.01	0.0505
**(2) GLM Model 2 (AIC: 348.2)**				
**Variables**	**Estimate**	**Std. Error**	**95% CI**	**Pr (>|z|)**
(Intercept)	−7.7356	0.4966	−8.71–−6.76	<0.001
c1	−5.3801	1.7344	−8.78–−1.98	0.0019
c3	7.4128	1.0195	5.41–9.41	<0.001
c5	4.8370	0.9627	2.95–6.72	<0.001
c7	−4.5296	1.9610	−8.37–−0.69	0.0209
**(3) GLM Model 3 (AIC: 359.8)**				
**Variables**	**Estimate**	**Std. Error**	**95% CI**	**Pr (>|z|)**
(Intercept)	−9.2392	0.3415	−9.91–−8.57	<0.001
c3	9.7219	0.7655	8.22–11.22	<0.001
c5	6.6416	0.9142	4.85–8.43	<0.001

**Table 3:** GLMM Model 3 (2N × WAIC: 209.3).

Variables	Coefficient	Stdev	95% Lower	95% Upper
(Intercept)	−10.4850	0.8257	−12.26	−9.05
c3	10.6013	2.2362	6.46	15.08
c5	5.8795	2.2257	1.49	10.35
r1_sd ^1^	1.7825	0.3193	1.23	2.48

^1^ The random factor of generalised linear mixture model (GLMM) by Bayesian inference was estimated based on a normal distribution (μ = 0, σ = 1.78). In the case of GLMM Model 3, 68.2% of the random factor value was in the range of ±1.78.
